# A novel nomogram based on cardia invasion and chemotherapy to predict postoperative overall survival of gastric cancer patients

**DOI:** 10.1186/s12957-021-02366-4

**Published:** 2021-08-28

**Authors:** Hanjun Mo, Pengfei Li, Sunfang Jiang

**Affiliations:** 1 Department of General Practice, Zhongshan Hospital, Fudan University, 111 Yixueyuan Road, Shanghai, 200032 China; 2 Department of General Surgery, Zhongshan Hospital, Fudan University, 180 Fenglin Road, Shanghai, 200032 China; 3 Health Management Center, Zhongshan Hospital, Fudan University, 180 Fenglin Road, Shanghai, 200032 China

**Keywords:** Nomogram, Gastric cancer, Overall survival, SEER

## Abstract

**Background:**

We aimed to establish and externally validate a nomogram to predict the 3- and 5-year overall survival (OS) of gastric cancer (GC) patients after surgical resection.

**Methods:**

A total of 6543 patients diagnosed with primary GC during 2004–2016 were collected from the Surveillance, Epidemiology, and End Results (SEER) database. We grouped patients diagnosed during 2004–2012 into a training set (*n* = 4528) and those diagnosed during 2013–2016 into an external validation set (*n* = 2015). A nomogram was constructed after univariate and multivariate analysis. Performance was evaluated by Harrell’s C-index, area under the receiver operating characteristic curve (AUC), decision curve analysis (DCA), and calibration plot.

**Results:**

The multivariate analysis identified age, race, location, tumor size, T stage, N stage, M stage, and chemotherapy as independent prognostic factors. In multivariate analysis, the hazard ratio (HR) of non-cardia invasion was 0.762 (*P* < 0.001) and that of chemotherapy was 0.556 (*P* < 0.001). Our nomogram was found to exhibit excellent discrimination: in the training set, Harrell’s C-index was superior to that of the 8th American Joint Committee on Cancer (AJCC) TNM classification (0.736 vs 0.699, *P* < 0.001); the C-index was also better in the validation set (0.748 vs 0.707, *P* < 0.001). The AUCs for 3- and 5-year OS were 0.806 and 0.815 in the training set and 0.775 and 0.783 in the validation set, respectively. The DCA and calibration plot of the model also shows good performance.

**Conclusions:**

We established a well-designed nomogram to accurately predict the OS of primary GC patients after surgical resection. We also further confirmed the prognostic value of cardia invasion and chemotherapy in predicting the survival rate of GC patients.

## Introduction

Gastric cancer (GC) remains the fifth most common cancer and the third main cause of cancer-related death, following lung cancer and colorectal cancer in both sexes [1]. More than one million people are diagnosed with GC annually, and the death toll is close to 800,000 [1]. The incidence among males is 2- to 3-fold higher than that among females (32.1 vs 13.2, per 100,000) in East Asia, whereas the rate in North America is generally low [1].

GC can be classified as cardia and non-cardia invasion, which have different epidemiology and causes [2, 3]. The incidence of non-cardia GC has declined over the past 30 years; however, cardia GC rates have remained stable or even increased [2, 4, 5]. The poor prognosis of cardia invasion compared to non-cardia has been reported [6, 7], but whether cardia invasion is an independent prognostic factor remains unknown.

Surgery is still the primary treatment to advanced GC [8], in which D2 lymphadenectomy has been widely carried out in Asia [9, 10]. A study from Japan of the 118,367 patients after surgical resection showed the 5-year overall survival (OS) rate is 71.1% [11]. However, recurrence occurs in approximately 20–50% of all patients after surgery [12]. Therefore, identifying prognostic factors is indispensable in choosing treatment methods and surveillance strategies.

A nomogram is one of the useful predictive tools for cancer due to its accuracy, practicability, and good discrimination [13]. It can quantify individual’s survival rate in graphic form and has been used for many tumors [14–16]. The classic nomogram for GC is the Memorial Sloan Kettering Cancer Center (MSKCC) nomogram created in 2003 [17]. Compared with the traditional staging system—the American Joint Committee on Cancer (AJCC) TNM classification, a nomogram incorporates more demographic and clinicopathologic factors into the model.

The 8th AJCC staging system was effective in 2018, but few studies have compared nomograms with this new edition. In addition, the role of chemotherapy in the prognosis of GC has been mentioned, but no nomograms have included chemotherapy as a variable to date [9, 18]. Finally, most of the established nomograms for GC are complicated or internally validated, or they have a small training set [9, 12, 18–20]. Consequently, we aim to establish and externally validate a relatively simple, generalized nomogram to predict the overall survival (OS) of primary GC patients after surgical resection. We hope to determine the value of identifying GC as cardia or non-cardia invasion while exploring the role of adjuvant chemotherapy. The performance of the nomogram is also compared with the AJCC 8th staging system.

## Materials and methods

### Patients and data set

**Fig. 1:**
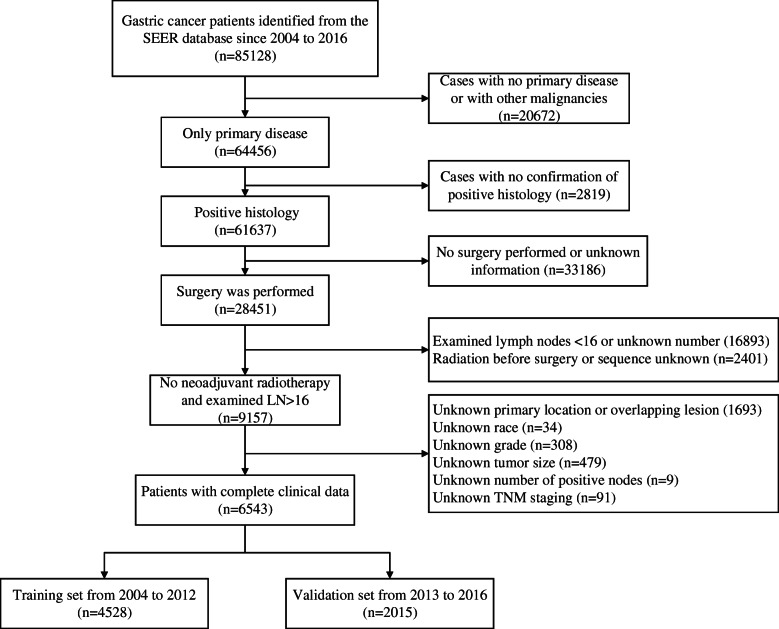
Enrollment flow chart of patient with primary GC after surgical resection according to inclusion and exclusion criteria. GC, gastric cancer; SEER, Surveillance, Epidemiology and End Results; LN, lymph node; TNM, tumor-node-metastasis

Data from patients diagnosed with primary GC during 2004–2016 were collected from the Surveillance, Epidemiology, and End Results (SEER) 18 Regs Custom Data Set (with additional treatment fields, Nov 2018 Sub), covering 27.8% of the US population [21]. The identification of GC patients was based on ICD-O-3/WHO 2008 histology codes. TNM staging was recoded according to the 8th AJCC TNM classification. The inclusion criteria were as follows: primary GC after surgical resection; no other malignancies; positive histology affirmation; no preoperative radiotherapy; more than 16 examined lymph nodes (LNs); and complete clinical data without missing values. The detailed enrollment process is presented in Fig. 1. Types of overlapping lesions and unspecified lesions were excluded. Finally, a total of 6543 cases were included in our study. We grouped them into a training set (*n* = 4528) and an external validation set (*n* = 2015) according to the year of diagnosis (2004–2012 and 2013–2016, respectively). Comparisons of demographic and clinicopathologic variables between the training and validation sets were generated using the “table1” function in R software.

### Construction of the nomogram

The cutoff values of continuous variables were determined using X-tile software designed by the Yale School of Medicine and our clinical experience. We divided patients into two groups according to their age (< 70 or ≥ 70 years) and into three groups according to the tumor size (< 2 cm, 2–10 cm, or ≥ 10 cm/diffuse). In variable of race, “other” included American Indian/AK Native and Asian/Pacific Islander. The SEER database classifies tumor histology (grade) into 4 groups: well differentiated (grade I), moderately differentiated (grade II), poorly differentiated (grade III), and undifferentiated/anaplastic (grade IV). We integrated poorly differentiated and undifferentiated/anaplastic tumors into a single group (named as “Poorly”) [21]. Location was further stratified into cardia and non-cardia invasion (including fundus, body, antrum and pylorus, lesser and greater curvature).

After univariate and multivariate analyses, independent prognostic factors were identified by the forward stepwise selection method. The proportional hazards (PH) assumption was examined before the multivariate analysis to ensure that the variables fitted the PH assumption. In the univariate analysis, the variables were further analyzed with the Cox proportional hazards (PH) regression model when *P* < 0.1. A nomogram was then constructed to predict the 3- and 5-year OS for primary GC patients after surgery. Kaplan-Meier (KM) survival curves were constructed and compared with the log-rank test.

### Nomogram performance

The performance of our nomogram was evaluated by discrimination and calibration. Discrimination was evaluated using Harrell’s C-index. The principle of the C-index has been described by Han et al. [9]. The *P*-value comparison of our nomogram with the AJCC staging system was achieved using the “compareC” function in R. The prediction was further evaluated by the area under the receiver operating characteristic curve (AUC) and the net benefit of decision curve analysis (DCA). Calibration was carried out by comparing the means of the nomogram-predicted survival rate with the actual OS measured by the KM method. Bootstraps were set to 1000 reiterations. Predicted total points were added as a new variable to the established nomogram in order to achieve external validation. Calibration plots of 3- and 5-year survival in the training set and 3-year survival in the validation set were constructed.

### Statistical analysis

Statistical analysis was performed using SPSS version 22.0 (SPSS, Chicago, IL, USA) and R 4.0.1 via rms, survival, table1, compareC, and ggplot2 packages. All tests were two-sided, and *P*-value < 0.05 was considered statistically significant. This study did not require local ethics approval.

## Results

### Patient characteristics

Demographic and clinicopathologic characteristics are presented in Table 1. The median age at diagnosis in both sets was 66 years, and male patients were the majority (60.5% and 61.3% in the training and validation sets, respectively). Cardia GC accounted for 25.1% of the whole GC population in the training set. Most of the patients (72.2% and 71.0%, respectively) had poorly differentiated disease. The median numbers of examined LNs were 23 (range, 19–31) and 24 (range, 19–33) in the training and validation sets, respectively. 33.1% of the patients received radiotherapy, and 52.1% received chemotherapy in the training set.

**Table 1:** Demographic and clinicopathologic variables of the training and validation sets

Variable	Training set (*n* = 4528)		Validation set (*n* = 2015)	
	*n*	%	*n*	%
Age (years)				
Median (range)	66 (55–75)		66 (56–74)	
< 70	2748	60.7	1226	60.8
≥ 70	1780	39.3	789	39.2
Sex				
Male	2741	60.5	1236	61.3
Female	1787	39.5	779	38.7
Race				
White	2787	61.6	1156	57.4
Black	568	12.5	263	13.1
Other	1173	25.9	596	29.6
Location				
Cardia	1135	25.1	364	18.1
Non-cardia	3393	74.9	1651	81.9
Grade (histology)				
Well	155	3.4	102	5.1
Moderately	1106	24.4	483	24.0
Poorly	3267	72.2	1430	71.0
AJCC 8th Stage				
I	1109	24.5	517	25.7
II	1513	33.4	501	24.9
III	1401	30.9	846	42.0
IV	505	11.2	151	7.5
T stage				
T1	736	16.3	448	22.2
T2	1676	37.0	251	12.5
T3	1407	31.1	727	36.1
T4	709	15.7	589	29.2
N stage				
N0	1274	28.1	717	35.6
N1	1176	26.0	359	17.8
N2	1054	23.3	353	17.5
N3	1024	22.6	586	29.1
M stage				
M0	4023	88.8	1865	92.6
M1	505	11.2	150	7.4
Tumor size (cm)				
< 2	476	10.5	328	16.3
2–10	3620	79.9	1532	76.0
≥ 10/diffuse	432	9.5	155	7.7
Examined LNs (No.)				
Median (range)	23 (19-31)		24 (19-33)	
Radiation				
No radiation or surgery	3029	66.9	1526	75.7
Yes	1499	33.1	489	24.3
Chemotherapy				
No/unknown	2167	47.9	873	43.3
Yes	2361	52.1	1142	56.7

*LN* lymph node, *AJCC* American Joint Committee on Cancer

### Analysis and development of the nomogram

Selected variables and hazard ratios (HRs) after univariate and multivariate analyses are listed in Table 2. We identified age, race, location, T stage, N stage, M stage, tumor size, and chemotherapy as independent prognostic factors associated with OS for GC patients. Due to a lack of significance, sex was excluded from the Cox PH regression model (HR, 0.973; 95% CI, 0.903–1.049).

**Table 2:** Variables associated with OS according to the Cox PH regression model

Variable	Univariable analysis			Multivariable analysis		
	HR	95% CI	*P-*value	HR	95% CI	*P*-value
Age, years						
< 70	Ref			Ref		
≥ 70	1.526	1.419–1.642	< 0.001	1.671	1.546–1.806	< 0.001
Sex						
Male	Ref					
Female	0.973	0.903–1.049	0.476			
Race						
White	Ref			Ref		
Black	1.043	0.934–1.163	0.456	1.079	0.965–1.207	0.183
Other	0.806	0.738–0.880	< 0.001	0.857	0.783–0.939	0.001
Location						
Cardia	Ref			Ref		
Non-cardia	0.906	0.835–0.983	0.018	0.762	0.699–0.831	< 0.001
Grade						
Well	Ref			Ref		
Moderately	1.515	1.182–1.942	0.001	0.972	0.756–1.249	0.824
Poorly	2.064	1.624–2.623	< 0.001	1.153	0.902–1.474	0.257
T stage						
T1	Ref			Ref		
T2	2.903	2.515–3.349	< 0.001	2.04	1.741–2.389	< 0.001
T3	3.896	3.371–4.504	< 0.001	2.265	1.919–2.674	< 0.001
T4	5.31	4.547–6.201	< 0.001	2.683	2.242–3.210	< 0.001
N stage						
N0	Ref			Ref		
N1	2.051	1.828–2.302	< 0.001	1.981	1.749–2.243	< 0.001
N2	3.527	3.147–3.952	< 0.001	3.308	2.912–3.758	< 0.001
N3	5.446	4.861–6.102	< 0.001	4.431	3.876–5.065	< 0.001
M stage						
M0	Ref			Ref		
M1	2.891	2.611–3.200	< 0.001	1.888	1.698–2.099	< 0.001
Tumor size, mm						
< 2	Ref			Ref		
2~10	2.477	2.122–2.892	< 0.001	1.158	0.980–1.369	0.085
≥ 10/diffuse	4.632	3.861–5.558	< 0.001	1.57	1.289–1.913	< 0.001
Chemotherapy						
No/unknown	Ref			Ref		
Yes	0.936	0.870–1.006	0.073	0.556	0.513–0.602	< 0.001

*HR* hazard ratio, *CI* confidence interval, *Ref* reference

**Fig. 2:**
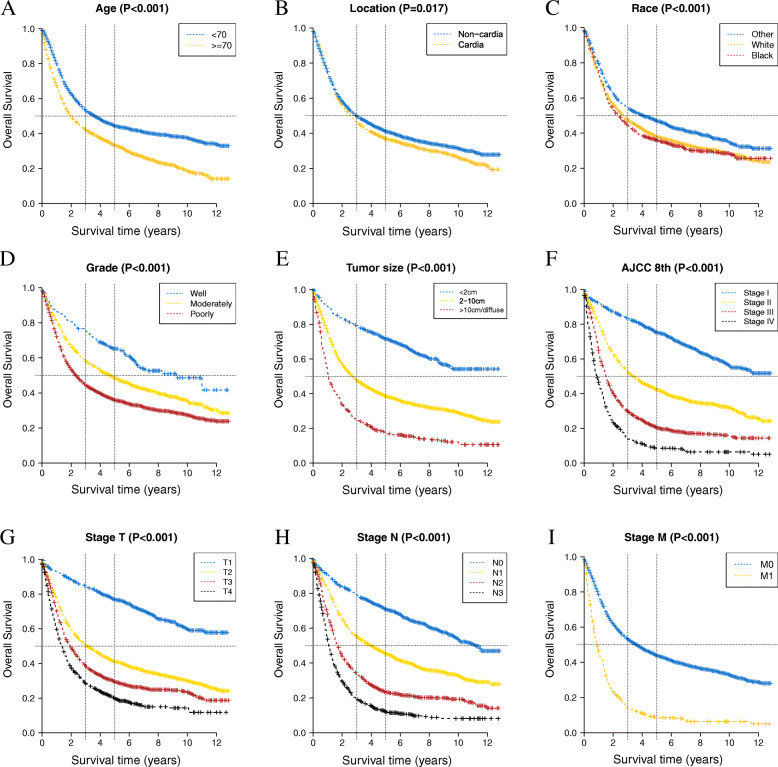
Kaplan-Meier survival curves of the independent prognostic factors (**A**–**E**, **G**–**I**) and TNM classification (**F**). The horizontal dotted line indicates the median survival time (OS = 0.5); the vertical line indicates the time at 3 and 5 years. AJCC, American Joint Committee on Cancer

**Fig. 3:**
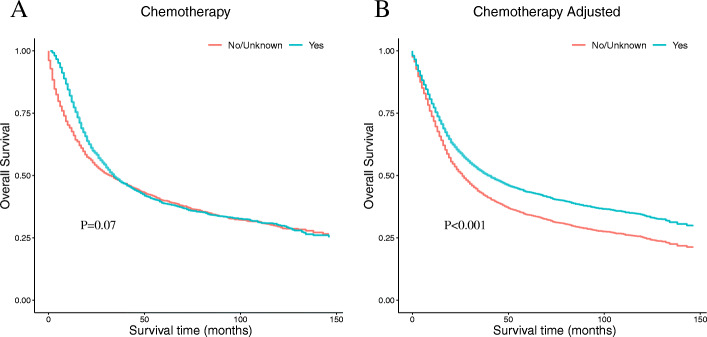
Kaplan-Meier survival curves of chemotherapy (**A**) and chemotherapy after adjustment (**B**)

Among the patients included in our research, HRs were found to be significantly higher for individuals who had the following characteristics: older than 70, male, black, cardia invasion, poorly differentiated disease, deeper invasion, more lymph node (LN) metastasis, distant metastasis, larger tumor size, and without chemotherapy. Of note, after adjustment for the multivariate analysis, the HR for location was 0.762 (95% CI, 0.699–0.831, *P* < 0.001), indicating that non-cardia invasion is an independent protective factor for GC prognosis. There are two distinct discrepancies between the univariate and multivariate analyses. Although grade was statistically significant in the univariate analysis, it seemed to be nonsignificant when adjusted by the multivariate model. Considering that grade represents histologic differentiation and is of clinical value, we still included it in the model. For chemotherapy, no statistically significant difference was observed in the univariate analysis (HR, 0.936; 95% CI, 0.870–1.006, *P* = 0.073). However, in the multivariate analysis, the difference became significant (HR, 0.556; 95% CI, 0.513–0.602, *P* < 0.001). The KM survival curves of select factors are shown in Figs. 2 and 3. In each panel of Fig. 2, the curves show good prognostic stratification for selected variables. As shown in Fig. 3, postoperative chemotherapy significantly prolonged patient survival after the adjustment for the Cox PH model.

**Fig. 4:**
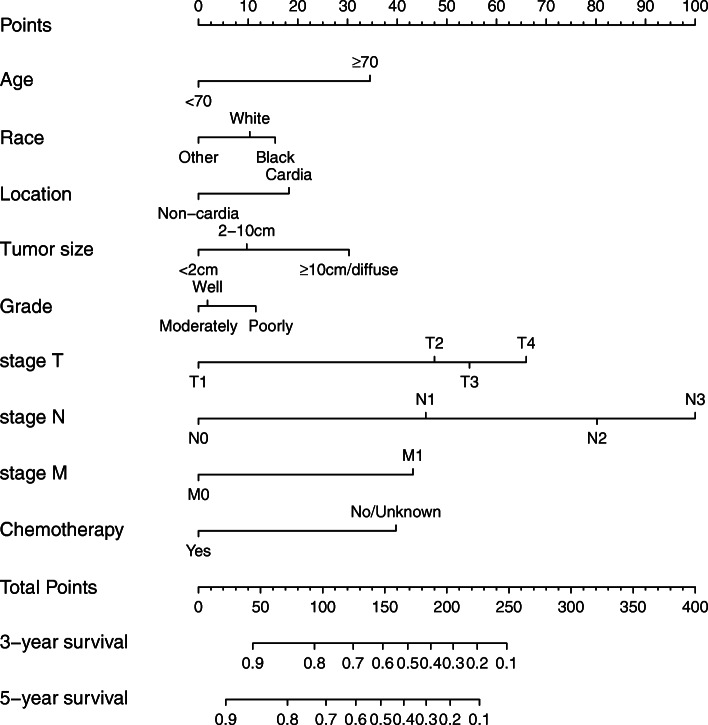
Nomogram predicting 3- and 5-year OS after surgical resection of GC. OS, overall survival; GC, gastric cancer; Other, American Indian/AK Native and Asian/Pacific Islander; Well, well differentiated; Moderately, moderately differentiated; Poorly, poorly differentiated and undifferentiated/anaplastic

The nomogram used to predict 3- and 5-year OS is shown in Fig. 4. From Fig. 4, we can see that *N* stage accounts for a large proportion of the total scores, indicating that the number of metastatic LNs is the most critical prognostic factor for GC. Patients with cardia invasion receive nearly 20 points, and those who do not undergo chemotherapy receive approximately 40 points.

### Performance of the nomogram

**Fig. 5:**
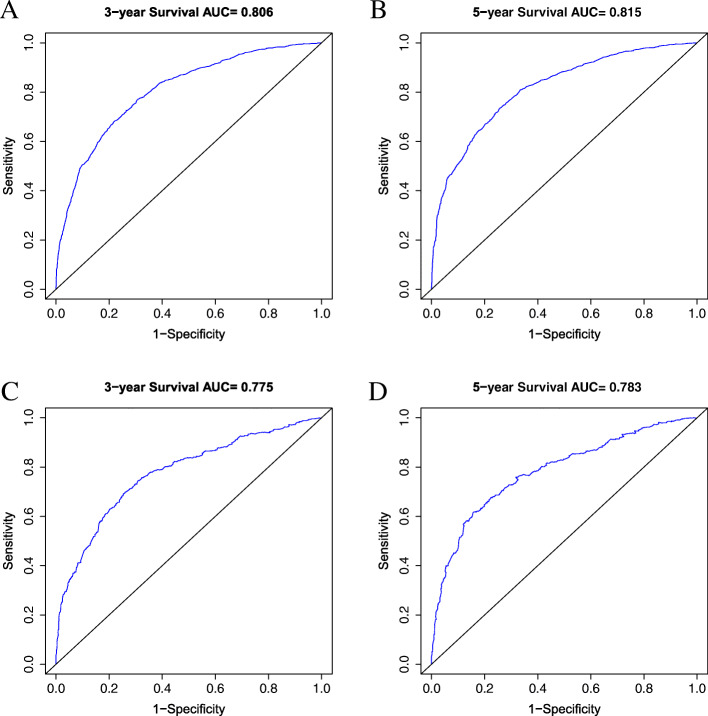
Receiver operating characteristic (ROC) curves predicting OS. **A** 3-year in the training set, **B** 5-year in the training set, **C** 3-year in the validation set, and **D** 5-year in the validation set. OS, overall survival; AUC, area under the receiver operating characteristic curve

**Fig. 6:**
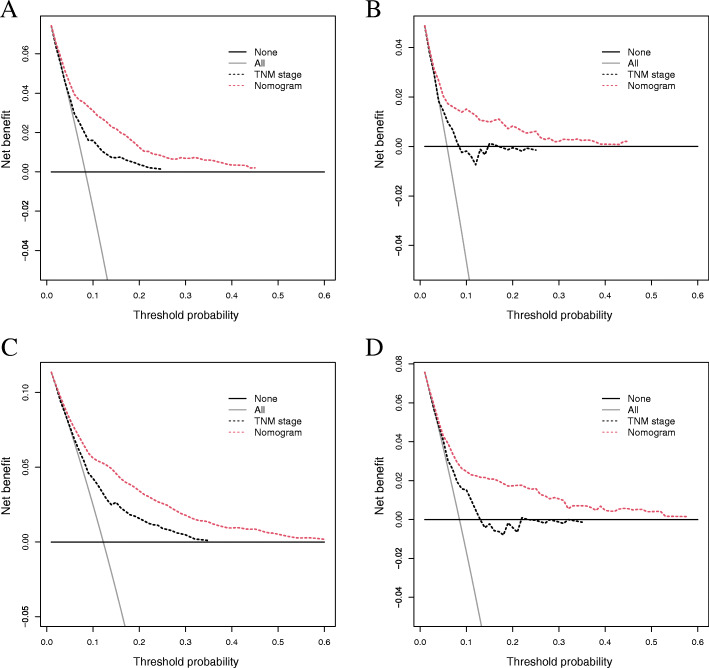
Decision curve analysis (DCA) to evaluate our nomogram and AJCC TNM classification. **A** 3-year and **B** 5-year in the training set; **C** 3-year and **D** 5-year in the validation set. Horizontal black line: no patients will die; inclined gray line: all patients will die. AJCC, American Joint Committee on Cancer; TNM, tumor-node-metastasis

In the training set (Table 3), the C-index was 0.736 (95% CI, 0.726–0.746), which was superior to that of the 8th AJCC TNM classification (C-index, 0.699; 95% CI, 0.689–0.709, *P* < 0.001). In the validation set, the C-index was also better (0.748 vs 0.707; 95% CI, 0.726–0.770 vs 0.684–0.730, *P* < 0.001). In addition, the AUCs of the nomogram exhibited great predictive ability in both the training and validation sets, with AUCs of 0.806 and 0.815 at 3 years and 5 years in the training set, respectively (Fig. 5A, B). In the validation set (Fig. 5C, D), the AUCs were only slightly reduced (0.775 and 0.783 for 3- and 5-year OS, respectively). The DCA results further demonstrated the good performance of our nomogram (Fig. 6). Regardless of the training (Fig. 6A, C) or validation set (Fig. 6B, D), our nomogram had a larger net benefit than the AJCC TNM classification. This favorable effect remains across a threshold probability of 0.05 to 0.45 for 3 years and 0.6 for 5 years.

**Table 3:** C-indexes for the nomogram and the AJCC 8th staging system in GC patients

OS	Training set		Validation set	
	C-index (95% CI)	*P*-value	C-index (95% CI)	*P*-value
Nomogram	0.736 (0.726–0.746)		0.748 (0.726–0.770)	
8th AJCC stage	0.699 (0.689–0.709)	< 0.001	0.707 (0.684–0.730)	< 0.001

*AJCC* American Joint Committee on Cancer, *GC* gastric cancer, *OS* overall survival, *CI* confidence interval

**Fig. 7:**
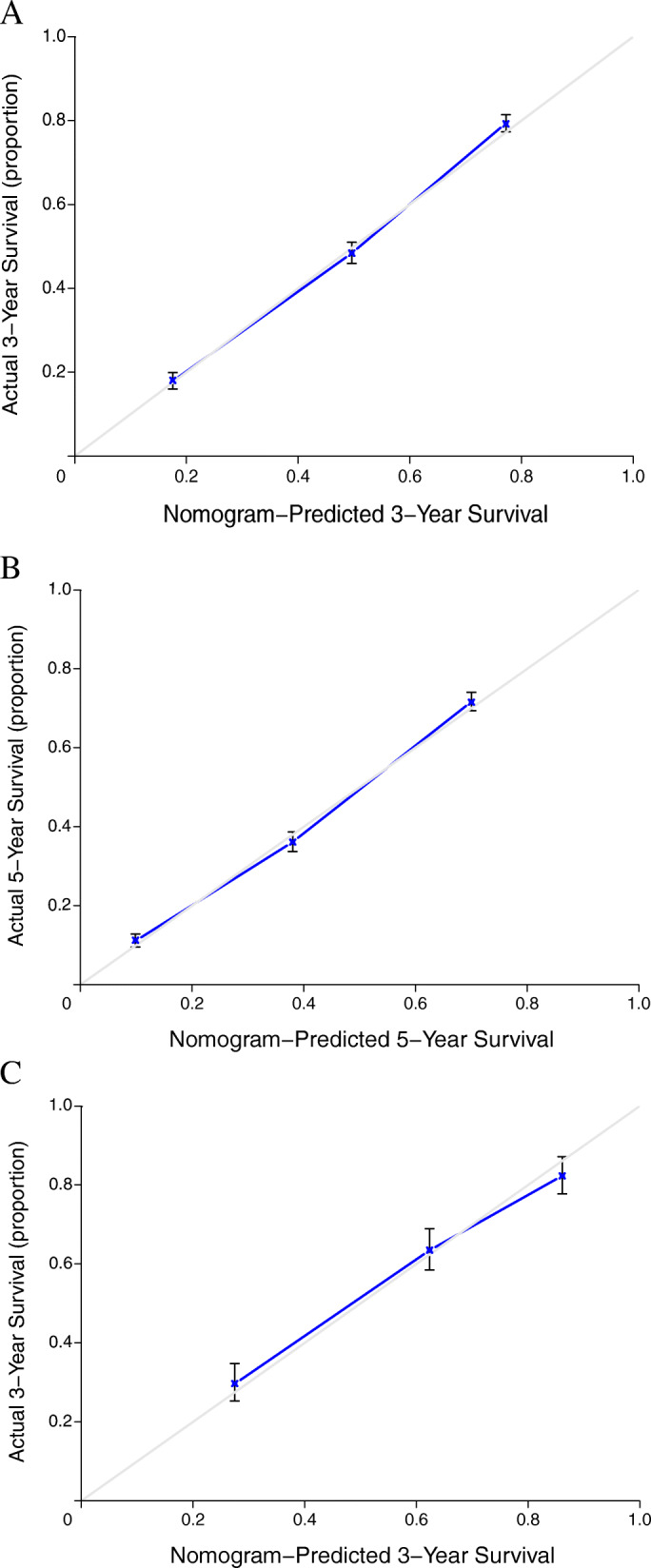
Calibration curves predicting overall survival. **A** 3-year in the training set, **B** 5-year in the training set, and **C** 3-year in the validation set. The *x*-axis represents nomogram-predicted survival; the *y*-axis represents actual survival and 95% confidence intervals (CIs) measured by Kaplan-Meier analysis

The calibration plots also showed good agreement for the nomogram-predicted 3-, 5-year survival in the training set and 3-year survival in the validation set (Fig. 7). The 5-year curve in the validation set cannot be constructed because of inadequate follow-up time (patients were diagnosed during 2013–2016). The diagonal line represents the ideal situation, and we can see that the predicted survival corresponds closely with the actual OS.

## Discussion

In the current study, we developed and externally validated a nomogram to predict 3- and 5-year OS for primary GC patients after surgical resection. We identified age, race, location, tumor size, T stage, N stage, M stage, and chemotherapy as independent prognostic factors, among which the number of metastatic LNs held the most weight [22]. Compared with the 8th AJCC TNM classification, our nomogram performed better in both the training and external validation sets.

Some nomograms classified GC location into the upper, middle, and lower third [9, 18]. In this study, we classified GC according to cardia invasion or no cardia invasion (the survival curves of the middle third and lower third were similar in our cohort; data not shown). As a result, we found that cardia GC had a worse prognosis than non-cardia GC (*P* < 0.001). Our finding is consistent with a systematic review, which found that patients with upper third GC had significantly increased all-cause mortality [23]. And when the gastroesophageal junction (GEJ) was excluded, the prognosis of pure cardia GC was even worse. Our data also showed that sex was not an independent prognostic factor, which was inconsistent with previous findings [9, 12, 17, 18, 24]. Although males and females differed in terms of incidence rate, their prognoses appeared to be similar.

Previously, Kim et al. found that age had nonlinear effects on HR [12]. Another study also found that patients older than 70 years had the lowest 5-year OS, compared with younger and middle-aged patients [25]. Their results were consistent with our analysis using X-tile, so we chose to convert age into a categorical variable at 70. Although grade is closely associated with malignant behavior and distant metastasis, it did not seem to be an independent factor in our study. Therefore, when we performed multivariate analysis, the *P-*value became insignificant.

Another discrepancy in this study pertains to chemotherapy [26]. Recent studies have proven that adjuvant chemotherapy after surgery could benefit patients in terms of survival probability [27]. A meta-analysis showed that compared with surgery alone, fluorouracil-based postoperative adjuvant chemotherapy significantly reduced the mortality of GC patients [28]. Another phase III randomized controlled trial (RCT) revealed that chemotherapy using capecitabine plus oxaliplatin for half a year after D2 gastrectomy improved the 3-year disease-free survival of GC compared with surgery alone (74% vs 59%, HR 0.56, *P* < 0.001) [29]. The results of our multivariate analysis further demonstrated that chemotherapy acted as a protective factor against poor outcomes (Fig. 3). We believe that chemotherapy did not show statistical significance in the univariate analysis was largely due to some confounding factors, such as age, location, or TNM staging. To the best of our knowledge, we are the first to finally include chemotherapy in the nomogram construction of GC.

Consistent with most previous studies, we excluded patients with fewer than 16 examined LNs [9]. This helps to ensure surgical quality and prevent the stage migration effect [9, 30]. In our study, the median examined LN numbers were 23 and 24 in the training and validation sets, respectively.

Quite a few studies used a randomly assigned (data-splitting) method to create a validation set [9, 19, 20]. However, theoretically, this method accounts as an internal validation rather than an external validation, leading to sample wasting as well as insufficient power for evaluation. In contrast, our external validation set was established according to the year of diagnosis (training set, 2004–2012; validation set, 2013–2016), which would produce a more convincing result.

Notably, 655 patients had distant metastasis (M1) but underwent surgery. Among them, 58.6% (384/655) received chemotherapy and 15.1% (99/655) received radiotherapy. A growing number of studies have shown that patients with unresectable stage IV GC can achieve good survival outcomes if they undergo radical gastrectomy after responding to several combined chemotherapy regimens [31]. This novel strategy is called conversion surgery, a treatment approach in which initially unresectable tumors become curable after chemotherapy response. If R0 resection is achieved, conversion surgery can significantly improve the patient survival rate [31]. Therefore, we did not exclude such patients and hope that our nomogram can be used with these patients to predict OS after surgery. Nevertheless, this concept is still controversial, and current cancer guidelines do not recommend surgery for stage IV patients.

There are some striking strengths in our study. First, we used the SEER database, a standardized and relatively comprehensive database with a large sample size. Data from 2004 were collected, and more than 6000 patients were ultimately included in our study. Second, to the best of our knowledge, we are the first to classify GC according to cardia/non-cardia invasion in a nomogram and found good discrimination in survival outcomes. We are also the first to finally include chemotherapy in the nomogram for GC as an independent prognostic factor. Third, our nomogram is based on the existing 8th AJCC staging system, which makes the nomogram widely available and highly convenient for clinical application.

Our study also has some limitations that should be noted. First, patients who did not receive chemotherapy and those with missing information were included in the SEER database, which added difficulty in determining the value of chemotherapy. As a result, the actual role of chemotherapy in patients’ prognosis could have been underestimated. Second, we did not further divide T4 and N3 stages in our results because 893 cases had T4 or N3 stage but lacked specific details. This may have sacrificed some precision but simplified the model. Finally, some novel biomarkers were reported to be useful in predicting prognosis of GC, such as differential gene expression, which will be tested in future studies [32–35].

## Conclusions

In summary, we established and externally validated an elaborate nomogram to predict 3- and 5-year OS for primary GC after surgical resection. We believe that our nomogram can achieve accurate predictions among Western populations. Future studies are needed to further evaluate its performance and extend its applicability.

## Data Availability

The datasets supporting our conclusion can be acquired from SEER database (https://seer.cancer.gov/). All R codes used in this study can be accessed via Github (https://github.com/wdxmo/nomogram/find/main).

## Abbreviations

SEER: Surveillance, Epidemiology, and End Results
AJCC: American Joint Committee on Cancer
GC: Gastric cancer
OS: Overall survival
HR: Hazard ratio
LN: Lymph node
TNM: Tumor-node-metastasis
ROC: Receiver operating characteristic
AUC: Area under receiver operating character curve
DCA: Decision curve analysis
